# Robust Indoor Positioning with Hybrid WiFi RTT-RSS Signals

**DOI:** 10.3390/s26010284

**Published:** 2026-01-02

**Authors:** Xu Feng, Khuong An Nguyen, Zhiyuan Luo

**Affiliations:** Computer Science Department, Royal Holloway University of London, Egham TW20 0EX, UK; khuong.nguyen@rhul.ac.uk (K.A.N.); zhiyuan.luo@rhul.ac.uk (Z.L.)

**Keywords:** indoor positioning, WiFi fingerprinting, conformal prediction, WiFi round-trip time

## Abstract

In 2025, there is still no ubiquitous, accurate, infrastructure-free indoor positioning system. Among existing approaches, WiFi-based positioning is highly promising as it leverages existing infrastructure. However, its performance is severely affected by WiFi signal variability and environmental dynamics. Thus, this paper presents a novel approach that combines WiFi Round Trip Time and Received Signal Strength measurements with the Conformal Prediction (CP) framework to achieve robust uncertainty-aware indoor positioning. Our proposed method does not only accurately estimate the user position, but also provides two prediction regions: a rectangular region and a circular region. We systematically evaluate our method across three real-world testbeds, which achieves a positioning accuracy of 0.6 m, while generating prediction regions with theoretical coverage guarantees for circular regions and marginal coverage guarantees for rectangular regions. To the best of our knowledge, this is one of the first work to enable uncertainty quantification on top of state-of-the-art WiFi ranging signals.

## 1. Introduction

Accurate indoor positioning is a key enabler for modern smart environments, ranging from asset tracking, patient monitoring to emergency responses. However, Global Positioning System (GPS) has severe limitations in indoor environments because of signal attenuation, multipath propagation, and building penetration losses. In the literature, WiFi-based indoor positioning has emerged as a popular solution, leveraging ubiquitous WiFi infrastructure in commercial and residential buildings [1,2,3,4].

Traditional approaches primarily rely on WiFi Received Signal Strength (RSS) fingerprinting [5,6,7,8,9], which estimates location by matching real-time measurements to a pre-constructed radio map. Despite its popularity, RSS-based positioning suffers from significant variability caused by environmental dynamics, device heterogeneity, and temporal signal fluctuations, often resulting in positioning errors of several meters.

The IEEE 802.11mc standard [10] introduced WiFi Round-Trip Time (RTT) through a Fine Timing Measurement (FTM) protocol, enabling direct range measurements between mobile devices and access points with sub-meter accuracy potential. Unlike RSS-based approaches that rely on signal strength variations, RTT provides explicit distance information, making it particularly robust and sensitive to environmental dynamics. The integration of machine learning and deep learning techniques with RTT measurements has further enhanced positioning accuracy, with recent studies demonstrating meter-level or even sub-meter-level performance in complex indoor environments [11,12]. However, despite achieving impressive point estimation accuracy, these models fail to quantify prediction uncertainty or provide confidence bounds.

To address these challenges, this paper provides a rigorous and comprehensive examination of conformal prediction techniques for WiFi RTT-based indoor positioning, with comparative analysis of RSS integration strategies. Conformal prediction offers a distribution-free framework capable of producing statistically valid prediction intervals with guaranteed coverage [13,14,15,16]. In contrast to traditional probabilistic approaches that rely on restrictive modelling assumptions (e.g., Gaussianity or known noise characteristics) conformal prediction requires only exchangeability, making it particularly well suited to indoor positioning contexts where wireless signal behaviours are highly variable, environment-dependent, and often non-Gaussian.

Despite its theoretical appeal, the application of conformal prediction to WiFi RTT localisation introduces several practical and methodological challenges that remain insufficiently explored in the literature [2,17]. These challenges motivate the need for a systematic investigation into how conformal prediction can be adapted, calibrated, and evaluated within real-world WiFi RTT-based indoor positioning pipelines, especially when fusing RTT with RSS measurements.

In summary, this paper makes the following contributions:

- This paper offers a comprehensive analysis of conformal prediction techniques for WiFi-based indoor positioning utilising WiFi RTT signal measures, highlighting their ability to deliver robust uncertainty quantification and thereby improve the reliability of the indoor positioning system.
- We evaluate the inductive conformal predictor, and assess its effectiveness and influence on WiFi RTT-based indoor positioning systems, validating reliable uncertainty estimates for both RTT and RSS inputs, with particularly notable benefits for high-precision RTT signals.
- We systematically examine the integration of RTT and RSS signal measurements within conformal prediction frameworks, providing a thorough comparison of RTT-only, RSS-only, and fused RTT+RSS approaches.
- We conduct extensive experimental validation across multiple real-world challenging indoor testbeds with diverse environmental characteristics, from office room, apartment, to university building floor.
- To the best of our knowledge, this is the first work to introduce and empirically assess rectangular and circular prediction regions for 2D indoor positioning. Our results reveal how the geometry of prediction regions affects coverage guarantees and provide practical guidance for uncertainty-aware indoor positioning systems.

The remainder of this paper is structured as follows. Section 2 reviews related work; Section 3 presents the problem formulation and system architecture; Section 4 describes the WiFi RTT protocol and the conformal prediction framework; Section 5 details the experimental setup; and Section 6 concludes the paper.

## 2. Related Work

WiFi-based indoor positioning systems have demonstrated significant progress, with traditional RSS-based methods achieving metre-level accuracy through fingerprinting techniques [1,2,18]. WiFi RTT represents a more advanced WiFi signal measure that can achieve sub-meter level accuracy due to its reliance on more stable time-of-flight measurements [11,19,20,21]. This makes WiFi RTT more sensitive to environmental variations and less susceptible to multipath effects compared to RSS [12,22,23,24,25]. Consequently, WiFi RTT is widely used in hybrid WiFi-based indoor positioning and line-of-sight (LOS)/non-line-of-sight (NLOS) identifications [26,27,28,29,30]. However, despite these technological advances, a critical gap remains in current WiFi indoor positioning implementations: the lack of robust uncertainty quantification mechanisms that can provide confidence measures and reliability indicators for location estimates.

Recent research has begun addressing uncertainty quantification in WiFi-based positioning through diverse frameworks. A Conformal Prediction (CP)-based indoor positioning system was proposed in [31] that outperforms traditional methods like Naïve Bayes and W-KNN by up to 20% in accuracy while providing statistically valid confidence measures. This method was later extended with Kullback-Leibler divergence to achieve 16–25% error reduction. The measurement uncertainty from wireless anchors was utilised in [32] with an observability-based filter and potential-based path planning to minimize localization uncertainty. The study in [33] proposed the integration of uncertainty quantification methodologies into deep learning models for RSS-based indoor localisation to enhance system dependability and performance. An interval random analysis approach was proposed in [34] for uncertain WiFi-based indoor localisation that enhances accuracy by employing an interval random parameter lognormal shadowing model for radio map enhancement. The authors in [35] applied conformal prediction to WiFi RSSI-based indoor positioning, achieving statistically guaranteed coverage where prediction sets contain the true location with pre-specified probability. Researchers proposed DumbLoc in [36], a machine learning framework using WiFi RSS fingerprinting that achieved 94.15% floor prediction accuracy and an 8.45 m mean positioning error on the UJI dataset while demonstrating cross-dataset generalisability. A CP method was proposed in [37] for fingerprint-based indoor localisation using WiFi Channel State Information (CSI) and Bluetooth Low Energy (BLE) RSS signals, achieving rigorous statistical guarantees with minimal extra training cost.

Despite these developments, CP implementation in WiFi indoor positioning systems remains relatively rare, and critically, none of the existing research has investigated CP applications to WiFi RTT-based positioning systems. This represents a substantial gap given RTT’s superior baseline accuracy, where CP could potentially provide even more reliable confidence regions while maintaining statistical guarantees for the predictions.

## 3. Problem Statement and System Architecture

This section begins by introducing the problem formulation for the proposed reliable WiFi RTT-based indoor positioning system. It then provides an overview of the system architecture underlying the proposed approach.

### 3.1. Problem Formulation

To estimate the unknown position of a user device, the WiFi-based indoor positioning system makes use of the preprocessed WiFi RTT and RSS measurements collected from multiple Access Points (APs) at known, fixed locations. It then utilises a machine learning model to estimate the user’s location based on newly reported WiFi signal measures.

Consider a network of *N* access points with known, fixed positions. For each AP *i*, two types of WiFi signal measurements are available:

(1) $${\mathrm{RTT}}_{i}:\mathrm{Round-Trip}\;\mathrm{Time}\;\mathrm{measurement}\;\mathrm{from}\;\mathrm{AP}\;i;$$

(2) $${\mathrm{RSS}}_{i}:\mathrm{Received}\;\mathrm{Signal}\;\mathrm{Strength}\;\mathrm{measurement}\;\mathrm{from}\;\mathrm{AP}\;i.$$

The complete measurement vector is defined as:

(3) $$z={\left[{\mathrm{RTT}}_{1},{\mathrm{RSS}}_{1},{\mathrm{RTT}}_{2},{\mathrm{RSS}}_{2},\ldots ,{\mathrm{RTT}}_{N},{\mathrm{RSS}}_{N}\right]}^{T}.$$

Alternatively, the measurements can be partitioned into RTT and RSS components:

(4) $$z_{\mathrm{RTT}}={\left[{\mathrm{RTT}}_{1},{\mathrm{RTT}}_{2},\ldots ,{\mathrm{RTT}}_{N}\right]}^{T};$$

(5) $$z_{\mathrm{RSS}}={\left[{\mathrm{RSS}}_{1},{\mathrm{RSS}}_{2},\ldots ,{\mathrm{RSS}}_{N}\right]}^{T}.$$

To accurately estimate the user’s location u, a supervised machine learning model is employed to directly map measurements to position estimates:

(6) $$\hat{u}=h_{\theta }\left(z\right),$$

where $\hat{u}$ is the estimated position from the positioning model, $h_{\theta }$ is the leveraged machine learning with learnable parameters θ, z is the complete measurement vector containing both RTT and RSS data.

The model parameters θ are learned by minimising a loss function over a dataset of *m* labeled training samples:

(7) $$\theta^{*}=\arg\min_{\theta }\sum_{j=1}^{m}L\left(u_{j},h_{\theta }\left(z_{j}\right)\right),$$

where a typical choice for L is the root mean squared error (RMSE) defined below

(8) $$L\left(u,\hat{u}\right)={∥u-\hat{u}∥}_{2}=\sqrt{{\left(x-\hat{x}\right)}^{2}+{\left(y-\hat{y}\right)}^{2}},$$

where (x,y) is the ground truth location u of the user and $\left(\hat{x},\hat{y}\right)$ is the positioning estimation $\hat{u}$ generated by the machine learning model.

This data-driven approach enables the model to learn complex relationships between signal measurements and spatial positions while naturally accommodating the nonlinear characteristics of indoor signal propagation.

### 3.2. System Architecture

**Figure 1:**
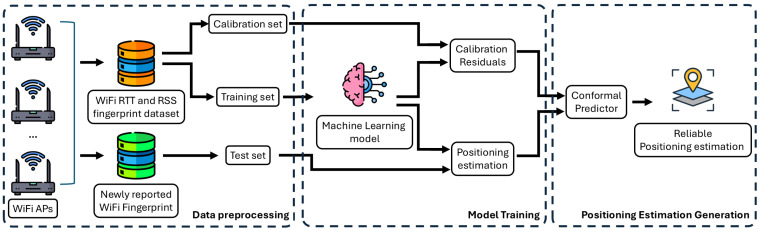
Overview of reliable WiFi-based Indoor Positioning Framework.

To deliver reliable WiFi-based indoor positioning estimates, the proposed framework adopts a comprehensive machine learning approach that integrates conformal prediction with WiFi fingerprinting, as show in Figure 1.

**Data preprocessing:** The system begins with data collection from multiple WiFi access points (APs) distributed throughout the indoor environment. By meticulously collecting the WiFi RTT and RSS signal measures at each location in the testbed, a WiFi fingerprint dataset is constructed. After preprocessing, including outlier removal and missing-value imputation, the collected dataset is split into training set and calibration set. These datasets are then fed into the machine learning model in the next stage.

**Model training:** At the core of the architecture lies the machine learning model, which learns to map WiFi fingerprint patterns to real-world geographic coordinates. During the training phase, the model processes the training set to identify complex patterns and correlations between WiFi signal characteristics and spatial locations. Next, the trained machine learning model is used to generate the positioning estimation for the calibration set. The residuals from these predictions, calculated as the differences between the predicted and ground truth locations in the calibration set, will then be used within the conformal prediction framework to provide reliable positioning estimation.

**Positioning estimation generation:** To ensure reliability, the architecture incorporates a conformal prediction module that quantifies the uncertainty of the estimated position. When WiFi RTT and RSS measurements are reported from an unknown user location, the machine learning model first generates a positioning estimate of the location. The conformal predictor then analyses the calibration residuals from the previous stage to construct prediction intervals at a user-specified confidence level. The final output is a reliability-aware positioning estimation, containing both the predicted location and its associated uncertainty bounds.

## 4. Reliable WiFi-Based Indoor Positioning Framework

This section provides a comprehensive description of the WiFi RTT protocol, WiFi RTT fingerprinting, and the conformal predictor employed in the proposed framework.

### 4.1. WiFi RTT and Fingerprinting

WiFi RTT represents a more advanced approach for indoor positioning that can achieve sub-meter level accuracy. Unlike RSS-based methods, RTT leverages the Fine Time Measurement (FTM) protocol to provide direct distance measurements by calculating the time-of-flight of WiFi signals traveling at the speed of light.

**Figure 2:**
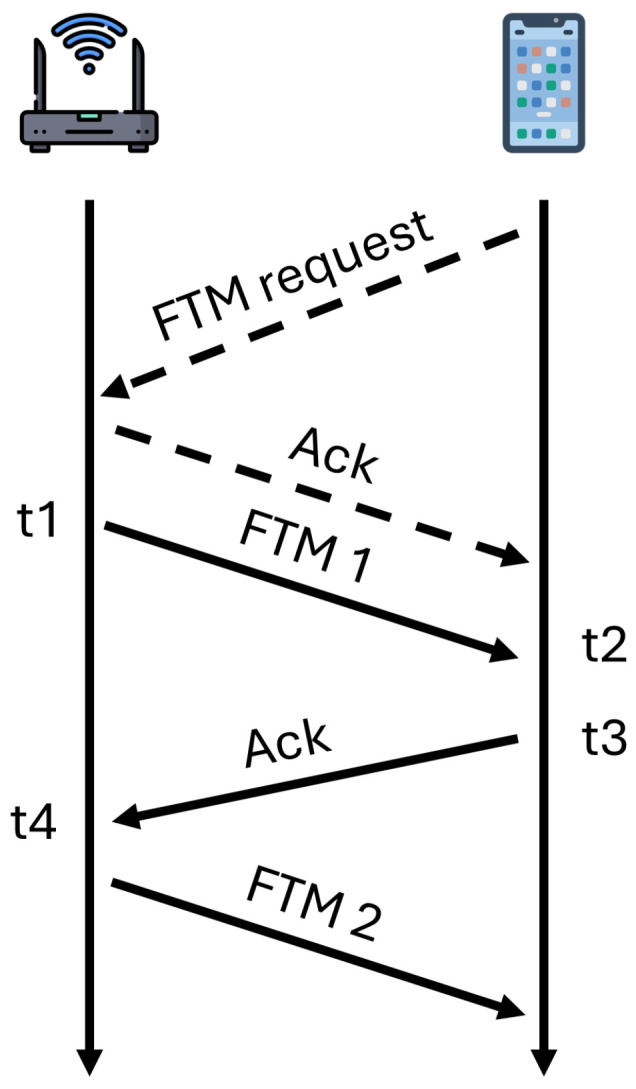
An overview of RTT protocol.

As shown in Figure 2, the initiation of RTT protocol is the transmission of a FTM request from the initiator (smartphone) to the responder (WiFi AP), specifying message count and intervals. Upon reception, the WiFi AP transmits a series of FTM messages, awaiting acknowledgment. The responder meticulously timestamps each FTM dispatch ($t_{1}$) and acknowledgment receipt ($t_{4}$), subsequently calibrating these timestamps using its internal clock. Simultaneously, the initiator acknowledges each FTM message, recording reception time ($t_{2}$) and acknowledgment transmission ($t_{3}$). Exchange of these temporal details allows both parties to calculate the round trip time, propagation time, and therefore the distance $D_{RTT}$ between the smartphone and WiFi AP is defined as

(9) $$D_{RTT}=\frac{\left(t_{4}-t_{1}\right)-\left(t_{3}-t_{2}\right)}{2}\times c,$$

where *c* is the speed of light.

While the RTT protocol provides a direct measure of the distance to the AP, it can be further complemented by fingerprint-based positioning. Although fingerprinting was originally developed using WiFi RSS measurements [4,38,39,40,41,42], recent studies have demonstrated that it can be seamlessly extended to incorporate RTT measurements as well [26]. In a typical WiFi fingerprinting workflow, two phases are involved: an offline phase and an online phase. During the offline phase, a comprehensive fingerprint dataset is constructed by collecting WiFi RTT and RSS signal measures, together with the corresponding ground-truth coordinates of reference locations.

After that, preprocessing methods are applied to the raw WiFi signal measures. To ensure the completeness of the fingerprinting dataset, more than 140 samples were collected at each reference point, although only 120 samples per location were required for the dataset utilised in the paper. After removing low-quality data samples that contain missing values, the WiFi data samples are labeled with the ground-truth coordinates of the reference points where they were collected. Next, to indicate WiFi measurements from APs that are too far away or not visible at current reference point, an RSS value of −200 dBm and an RTT value of 100,000 mm are assigned (see Section 5.1).After appropriate preprocessing, this dataset is used to train a machine learning Random Forest model. For all Random Forest models leveraged in this study, several model parameters were chosen from predefined ranges, including the number of estimators, maximum tree depth, etc. Multimodal models employed moderately deep trees with fewer estimators, whereas unimodal models favoured a larger ensemble with shallower trees to balance bias and variance.

In the online phase, when a user enters the testbed at an unknown location, a new WiFi data sample is captured and compared against the stored fingerprints in the dataset. Finally, the trained machine learning model produces an estimate of the user’s current location.

### 4.2. Uncertainty Quantification via Conformal Prediction

Although machine learning models can estimate user locations using pre-constructed WiFi fingerprinting datasets, they often lack reliable measures of the positioning estimation uncertainty. To address this, conformal prediction is employed, offering a rigorous, distribution-free framework for generating positioning estimation regions with statistical guarantees.

To reliably quantify uncertainty in WiFi-based indoor positioning, our goal is to construct prediction regions *C* that contain the true user position with a specified probability (see Algorithm 1), denoted as:

(10) $$P(u_{\mathrm{new}}\in C\left(z_{\mathrm{new}}\right))\geq 1-\epsilon ,$$

where $u_{\mathrm{new}}$ is the ground truth coordinates of the user’s current location, $C(z_{\mathrm{new}})$ is the predicted region under user-specified confidence level, $z_{\mathrm{new}}$ is the newly reported WiFi signal measures collected by the user, ϵ is the desired miscoverage rate, 1−ϵ is the confidence level representing the probability that the prediction region contains the true user position.

To generate the predicted region, the WiFi fingerprint dataset is first split into training set and calibration set. Next the machine learning-based positioning model $h_{\theta }$ is trained the training set obtain optimal parameters $\theta^{*}$. After training, the machine learning model $h_{\theta^{*}}$ is applied to the calibration set. For each calibration example, the Euclidean distance between the true position and the model’s prediction is calculated, serving as the nonconformity score, defined as:

(11) $$r_{i}={∥u_{i}-h_{\theta^{*}}\left(z_{i}\right)∥}_{2},\;i=m+1,\ldots ,m+n.$$

where $r_{i}$ is the residual (i.e., nonconformity score $\alpha_{i}$) of the *i* th sample in the calibration set, $u_{i}$ and $z_{i}$ are the ground truth location and WiFi signal measurements of the sample, respectively, *m* is the total number of training samples and *n* is the total number of calibration samples. The nonconformity score will be used to quantify how unusual a true location is given the measurements and the model’s prediction.
**Algorithm 1** Robust Indoor Positioning with Hybrid WiFi RTT-RSS Signals.  1: **Input:**
      $D={\left\{\left(z_{i},u_{i}\right)\right\}}_{i=1}^{m+n}$: WiFi fingerprint dataset      $z_{i}={\left[{\mathrm{RTT}}_{1},{\mathrm{RSS}}_{1},\ldots ,{\mathrm{RTT}}_{N},{\mathrm{RSS}}_{N}\right]}^{T}$: WiFi measurements from *N* APs      $u_{i}=\left(x_{i},y_{i}\right)$: ground truth coordinates      *m*: number of training samples      *n*: number of calibration samples      ϵ∈(0,1): miscoverage rate      $z_{\mathrm{new}}$: new WiFi measurement  2: **Output:**
      ${\hat{u}}_{\mathrm{new}}=\left({\hat{x}}_{\mathrm{new}},{\hat{y}}_{\mathrm{new}}\right)$: point estimate      $C_{\mathrm{rect}},C_{\mathrm{circ}}$: prediction regions with $P(u_{\mathrm{new}}\in C)\geq 1-\epsilon$  3:   4: $D_{\mathrm{train}}\leftarrow {\left\{\left(z_{i},u_{i}\right)\right\}}_{i=1}^{m}$  5: $\theta^{*}\leftarrow \arg\min_{\theta }\sum_{j=1}^{m}{∥u_{j}-h_{\theta }\left(z_{j}\right)∥}_{2}$  6: $D_{\mathrm{cal}}\leftarrow {\left\{\left(z_{i},u_{i}\right)\right\}}_{i=m+1}^{m+n}$  7: **for** i=m+1 to m+n **do**  8:       ${\hat{u}}_{i}=\left({\hat{x}}_{i},{\hat{y}}_{i}\right)\leftarrow h_{\theta^{*}}\left(z_{i}\right)$  9:       $r_{x,i}\leftarrow \left|x_{i}-{\hat{x}}_{i}\right|$10:       $r_{y,i}\leftarrow \left|y_{i}-{\hat{y}}_{i}\right|$11:       $r_{p,i}\leftarrow \sqrt{{\left(x_{i}-{\hat{x}}_{i}\right)}^{2}+{\left(y_{i}-{\hat{y}}_{i}\right)}^{2}}$12: **end for**13: $q_{x}\leftarrow {\mathrm{Quantile}}_{1-\epsilon }\left(\left\{r_{x,m+1},\ldots ,r_{x,m+n}\right\}\right)$14: $q_{y}\leftarrow {\mathrm{Quantile}}_{1-\epsilon }\left(\left\{r_{y,m+1},\ldots ,r_{y,m+n}\right\}\right)$15: $q_{p}\leftarrow {\mathrm{Quantile}}_{1-\epsilon }\left(\left\{r_{p,m+1},\ldots ,r_{p,m+n}\right\}\right)$16: ${\hat{u}}_{\mathrm{new}}=\left(\hat{x},\hat{y}\right)\leftarrow h_{\theta^{*}}\left(z_{\mathrm{new}}\right)$17: $C_{\mathrm{rect}}\leftarrow \{\left(x^{\prime },y^{\prime }\right):|x^{\prime }-\hat{x}|\leq q_{x},|y^{\prime }-\hat{y}\left|\leq q_{y}\right\}$18: $C_{\mathrm{circ}}\leftarrow \{\left(x^{\prime },y^{\prime }\right):∥\left(x^{\prime },y^{\prime }\right)-{\hat{u}}_{\mathrm{new}}{∥}_{2}\leq q_{p}\}$19: 20: **return** ${\hat{u}}_{\mathrm{new}},C_{\mathrm{rect}},C_{\mathrm{circ}}$

Once the residuals of the calibration set is calculated, the (1−ϵ)-quantile *q* of the calibration residuals is identified, defined as:

(12) $$q={\mathrm{Quantile}}_{1-\epsilon }\left(\left\{r_{m+1},\ldots ,r_{m+n}\right\}\right).$$

Thus, for any newly reported WiFi signal measurement $z_{\mathrm{new}}$, the prediction region is generated as:

(13) $$C\left(z_{\mathrm{new}}\right)=\left\{\bar{u}\in R^{2}:{∥\bar{u}-h_{\theta^{*}}\left(z_{\mathrm{new}}\right)∥}_{2}\leq q\right\},$$

where $C(z_{\mathrm{new}})$ is centered at the model’s point estimate, with radius *q*, $\bar{u}$ is the candidate location forming the predicted region. For the independent prediction of the *x*-coordinate, the quantity $q_{x}$ represents the half-width of the prediction interval $\left[\hat{x}-q_{x},\;\hat{x}+q_{x}\right]$.

## 5. Empirical Results

This section provides a thorough evaluation of the proposed reliable WiFi-based indoor positioning system. We begin by introducing the real-world datasets used in our study, followed by a detailed investigation of the empirical experimental results.

### 5.1. Experimental Setup and Data Collection

**Figure 3:**
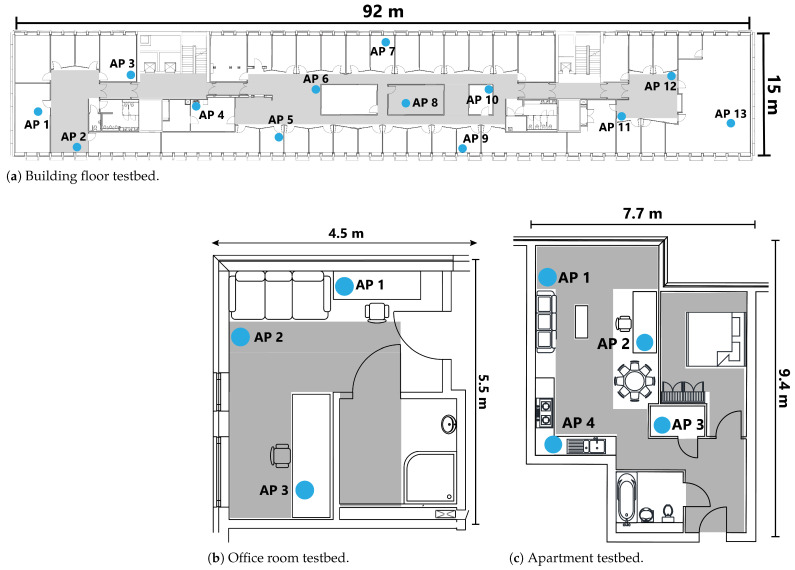
Layout of the 3 testbeds. The blue dots show the locations of the RTT-enabled APs. The placement of the APs replicates their real-world locations. All measurements are taken in the grey areas.

To evaluate the performance of the proposed reliable WiFi-based indoor positioning system, examine the robustness across devices and time and demonstrate the generalisation of the results, we conducted experiments in three representative and challenging real-world environments: a full floor of a campus building, an office room, and a residential apartment (see Figure 3) [26]. This dataset includes WiFi RTT and RSS measurements, along with line-of-sight (LoS) annotations for every reference point. These three datasets were collected over different time period in real-world complex scenarios that contain distinguishing LoS conditions. Each reference point comprises more than 120 WiFi scans, as shown in Table 1. A desktop PC equipped with an Intel Core i9-12900K processor (Intel Corporation, Santa Clara, CA, USA) and 32 GB DDR4 4000 MHz memory (G.SKILL International Enterprise Co., Ltd., Taipei, Taiwan) was used to analyse the results. On the largest building floor dataset, the model training time was 1.6 s, and the average generation time for each *q*-value was 0.2 s. These low computational requirements indicate that the proposed approach is lightweight and amenable to practical deployment.

In the Building Floor dataset, 13 RTT-enabled Google WiFi points (Google LLC, Mountain View, CA, USA) (see Table 2) were deployed to mirror their real-world positions within the building. WiFi data were collected using an LG G8X ThinQ smartphone (LG Electronics Inc., Seoul, Republic of Korea) (see Table 3). Please note that no human subjects were involved in the data collection. The smartphone was mounted on a tripod during all measurements at human chest height, and therefore no ethics approval or informed consent was required. Other WiFi RTT-enabled access points include the Google Nest WiFi Pro (Google LLC, Mountain View, CA, USA), Cisco 9164 (Cisco Systems, Inc., San Jose, CA, USA) and Aruba AP755 (HPE Aruba Networking, Santa Clara, CA, USA), among others. WiFi RTT-enabled smartphones include the Google Pixel 9, Samsung SM-S918B (Samsung Galaxy S23 Ultra) (Samsung Electronics Co., Ltd., Suwon, Republic of Korea) and Xiaomi Mi 10 Pro (Xiaomi Corporation, Beijing, China). A full list is available at https://developer.android.com/develop/connectivity/wifi/wifi-rtt#supported-aps (accessed on 29 December 2025).

Table 4 provides a snapshot of the dataset. Columns ’AP1 RSS’ to ’AP13 RSS’ contain the received signal strength from each AP, with −200 dBm denoting that the AP is not detected at the reference point. Columns X and Y give the ground-truth coordinates, and the LoS APs column specifies which APs have direct LoS. Table 4b illustrates the corresponding RTT data, where a value of 100,000 mm indicates the RTT measurement from unheard APs. For performance assessment, we ensured no overlap between training and testing locations.

### 5.2. Baseline Performance of WiFi-Based Indoor Positioning

**Figure 4:**
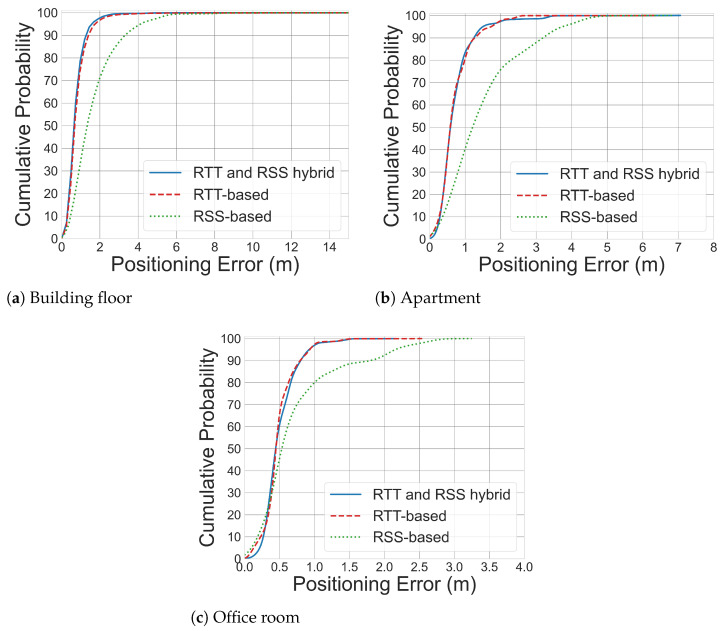
CDF curves of WiFi-based indoor positioning using different WiFi signal measurement on different testbeds. It is observed from the CDF curves that WiFi RTT-RSS fingerprinting delivers the best positioning estimation.

To evaluate the performance of the proposed reliable WiFi-based indoor positioning system, we employ a Random Forest (RF) model as the primary predictor, as it has been identified in the literature [26] as one of the most effective approaches for this task. As illustrated in Figure 4 and Table 5, the hybrid WiFi RTT and RSS-based indoor positioning system delivers the most promising positioning estimation, with an accuracy of 0.6 m for the challenging large-scale real-world Building Floor dataset. The positioning accuracy achieves 0.59 m and 0.38 m for Apartment and Office Room dataset, respectively. It is observed that, even in the complex building-floor dataset containing mixed line-of-sight (LOS) and non-line-of-sight (NLOS) conditions, the hybrid system that combines WiFi RTT and RSS measurements achieves a positioning accuracy below 1 m, 80% of the time.

### 5.3. Conformal Prediction for WiFi-Based Indoor Positioning

Traditional machine learning models, including the Random Forest predictor employed above, provide location estimations without confidence measures, making it difficult to assess the reliability of individual predictions or to identify when the system may be operating under challenging conditions. To address this gap, we apply CP on the three complicated real-world datasets to generate prediction intervals and regions with guaranteed coverage rates, providing quantifiable uncertainty estimates for location predictions.

Three distinct *q*-values for different aspects of location prediction are produced. The first $q_{x}$ is the half-width of the *x*-coordinate prediction interval and ensures that the true x-coordinate falls within $\left[\hat{x}-q_{x},\hat{x}+q_{x}\right]$ with probability 1−ϵ. Similarly, $q_{y}$ guarantees that the true *y*-coordinate falls within $\left[\hat{y}-q_{y},\hat{y}+q_{y}\right]$ with the same coverage level. The third value $q_{p}$, is designed for direct 2D positioning and generates a circular prediction region.

These three *q*-values produce two fundamentally different types of 2D prediction regions. The first type, derived from $q_{x}$ and $q_{y}$, creates a rectangular region defined as

(14) $$C_{rect}=\left\{\left(x^{\prime },y^{\prime }\right)|x^{\prime }\in \left[\hat{x}-q_{x},\hat{x}+q_{x}\right],y^{\prime }\in \left[\hat{y}-q_{y},\hat{y}+q_{y}\right]\right\}.$$

The second type, derived from $q_{p}$, produces a circular region defined as

(15) $$C_{circ}=\left\{\left(x^{\prime },y^{\prime }\right)|\sqrt{{\left(x^{\prime }-\hat{x}\right)}^{2}+{\left(y^{\prime }-\hat{y}\right)}^{2}}\leq q_{p}\right\}.$$

This dual approach allows us to examine how different geometric representations of uncertainty perform in practice. The choice of circular and rectangular prediction regions is motivated by the conformal prediction framework: circular regions arise naturally from the Euclidean distance nonconformity measure $r_{i}={∥u_{i}-{\hat{u}}_{i}∥}_{2}$, whilst rectangular regions result from independent marginal predictions for x and y coordinates, aligning with Cartesian building layouts. Both geometries support efficient real-time implementation and provide the necessary comparison to reveal the fundamental trade-off between coverage reliability and region size.

To evaluate the performance of conformal prediction within the indoor positioning framework, we assess two key aspects: **Coverage Rate** and **Efficiency**.

- **Coverage Rate**
  Coverage rate measures the empirical proportion of test instances in which their true values lie within the predicted intervals or regions. For a target confidence level of 1−ϵ, CP guarantees that the coverage rate
  $$\frac{1}{k}\sum_{i=1}^{k}1\left\{\mathrm{true}\;{\mathrm{value}}_{i}\in \mathrm{prediction}\;\mathrm{region}\;C_{i}\right\}$$
  is at least 1−ϵ under the assumption of exchangeability with *k* being the number of test samples, where 1 is an indicator function that takes the value of 1 if $\mathrm{true}\;{\mathrm{value}}_{i}\in$ $\mathrm{prediction}\;\mathrm{region}\;C_{i}$. In our experiments, four coverage metrics are reported:
  1. **$q_{x}$ Coverage:** proportion of test instances where the true *x*-coordinate lies within $\left[\hat{x}-q_{x},\;\hat{x}+q_{x}\right]$, where $q_{x}$ represents the half-width of the prediction interval.
  2. **$q_{y}$ Coverage:** proportion of test instances where the true *y*-coordinate lies within $\left[\hat{y}-q_{y},\;\hat{y}+q_{y}\right]$, where $q_{y}$ represents the half-width of the prediction interval.
  3. **2D Coverage:** proportion of test instances lying inside the rectangular region
    $$C_{rect}=\left\{\left(x^{\prime },y^{\prime }\right)|x^{\prime }\in \left[\hat{x}-q_{x},\hat{x}+q_{x}\right],\;y^{\prime }\in \left[\hat{y}-q_{y},\hat{y}+q_{y}\right]\right\}.$$
  4. **$q_{p}$ Coverage:** proportion of test instances lying within the circular region
    $$C_{circ}=\left\{\left(x^{\prime },y^{\prime }\right)|\sqrt{{\left(x^{\prime }-\hat{x}\right)}^{2}+{\left(y^{\prime }-\hat{y}\right)}^{2}}\leq q_{p}\right\}.$$
- **Efficiency**
  Efficiency quantifies the tightness or size of the prediction regions. Under identical coverage rates, smaller regions indicate more efficient predictions, meaning that the CP model provides more precise and confident estimates while still satisfying the required coverage guarantee.

**Figure 5:**
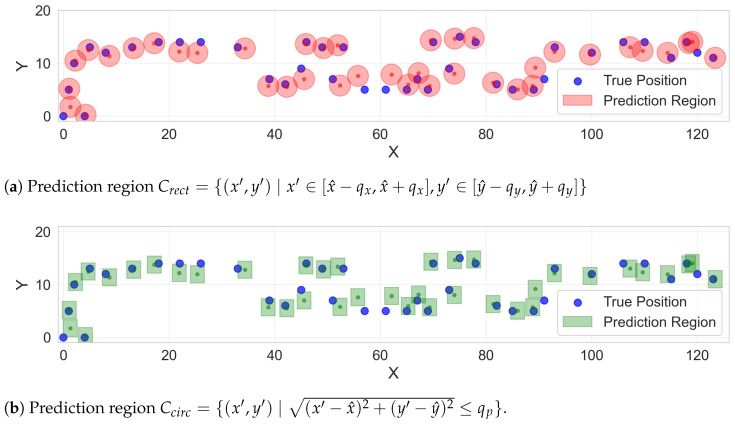
WiFi-based indoor positioning results using conformal prediction with 90% confidence level in building floor testbed. Blue points indicate ground truth locations, red circles represent prediction regions $C_{rect}=\left\{\left(x^{\prime },y^{\prime }\right)|x^{\prime }\in \left[\hat{x}-q_{x},\hat{x}+q_{x}\right],y^{\prime }\in \left[\hat{y}-q_{y},\hat{y}+q_{y}\right]\right\}$ and the green rectangles represent prediction regions $C_{circ}=\left\{\left(x^{\prime },y^{\prime }\right)|\sqrt{{\left(x^{\prime }-\hat{x}\right)}^{2}+{\left(y^{\prime }-\hat{y}\right)}^{2}}\leq q_{p}\right\}$ with guaranteed coverage rate. The area of the circular region (**a**) and the rectangular region (**b**) are 4.52 m^2^ and 3.10 m^2^, respectively. However, the actual coverage rates are 90.01% and 81.75%, respectively. These results indicate the trade-off between efficiency (prediction region area) and coverage rage in uncertainty quantification.

As shown in Table 6 and Table 7, across all three testbeds and all signal types, the proposed methods successfully achieve the target coverage rates for individual coordinates and circular regions ($q_{p}$ Coverage). The $q_{x}$ Coverage, $q_{y}$ Coverage, and $q_{p}$ Coverage metrics consistently meet or slightly exceed their target levels (90% or 95%), confirming that CP’s validity guarantee holds in practice for this application domain. As shown in Figure 5, the conformal prediction approach generates tight uncertainty regions (pink circles and green rectangles) around the true positions (blue dots), providing reliable coverage guarantees for indoor localisation.

However, while the circular regions ($q_{p}$ Coverage) successfully meets the target coverage rate, a systematic pattern emerges when examining the rectangular regions (2D coverage rates). These consistently fall below the target coverage levels, typically achieving 80–86% coverage when targeting 90%, and 90–94% coverage when targeting 95%. This phenomenon occurs because the rectangular region requires both x and y coordinates to simultaneously fall within their respective intervals. This occurs because the 2D rectangular region requires both x and y to fall within their respective intervals simultaneously, so the joint coverage becomes the product of the two marginal coverages. This finding highlights an important consideration when choosing between rectangular and circular prediction regions for 2D positioning applications.

The comparison across different WiFi signal types reveals a clear performance hierarchy that remains consistent across all three testbeds. The RTT + RSS hybrid approach demonstrates the best efficiency, producing the smallest *q*-value and thus the tightest prediction regions. For example, in the Building Floor testbed at 90% confidence, the hybrid approach achieves $q_{x}=0.79$ m. The RTT Only approach performs at a moderate level, producing regions 4–50% larger depending on the testbed. The RSS Only approach consistently produces the largest *q*-values, indicating substantially more uncertainty, with predictions 100–200% larger than the hybrid approach. This clear ordering demonstrates that combining RTT measurements with RSS provides complementary information that significantly improves both accuracy and certainty in position predictions.

The three testbeds exhibit distinct characteristics that reflect their physical environments. The smallest Office Room testbed consistently shows the tightest prediction regions across most methods, with the RTT+RSS hybrid approach at 90% confidence achieving $q_{p}=0.90$ m and 0.96 m at 95%. The Apartment testbed demonstrates comparable performance, with $q_{p}=1.11$ m at 90% and 1.52 m at 95% for the hybrid approach. The Building Floor testbed shows moderately larger prediction regions, with the hybrid approach achieving $q_{p}=1.20$ m at 90% and 1.45 m at 95%. Considering that the Building Floor dataset is a complex, large-scale testbed containing both LOS and NLOS conditions, these results remain promising, as the predictor is still able to provide reasonably tight uncertainty bounds despite the challenging signal propagation environment.

**Figure 6:**
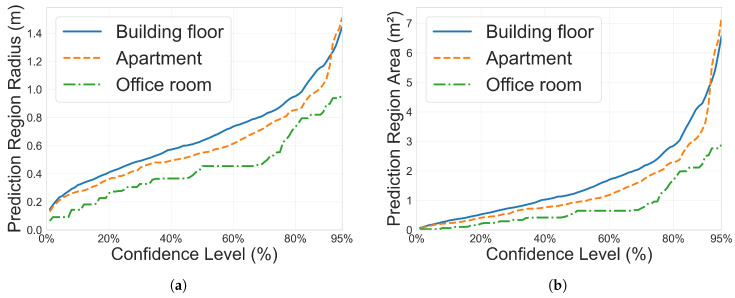
Circular prediction region radius (**a**) and area (**b**) as a function of the user-specified confidence level in different testbeds. Hybrid WiFi RTT and RSS measurements are selected as the input signal measures. It is observed from the curves that higher confidence levels always require larger prediction regions. (**a**) Circular prediction region radius as a function of the user-specified confidence level. (**b**) Circular prediction region area as a function of the user-specified confidence level.

Finally, the relationship between confidence level and prediction region size reveals the fundamental trade-off in uncertainty quantification, as shown in Figure 6 and Table 8. When increasing confidence from 90% to 95%, rectangular prediction regions grow substantially: for the hybrid approach, areas expand from 3.10 m^2^ to 4.78 m^2^ (Building Floor), 2.82 m^2^ to 5.06 m^2^ (Apartment), and 1.36 m^2^ to 2.69 m^2^ (Office Room), with similar growth patterns observed for circular regions and other signal types. More critically, a consistent trade-off emerges between region size and coverage reliability across all conditions. Rectangular regions consistently achieve smaller areas than circular regions. For example, at 90% confidence in the Building Floor testbed, the hybrid approach yields 3.10 m^2^ (rectangular) versus 4.52 m^2^ (circular). This efficiency advantage, representing 30–45% reduction in area, persists across different signal measures and testbeds. However, this spatial efficiency comes at the cost of coverage reliability. It is observed that rectangular regions systematically achieve 2D coverage rates 8–10 percentage points below target levels (e.g., 81.75% actual versus 90% nominal), while circular regions consistently meet or slightly exceed their specified confidence levels (e.g., 90.01% for 90% target). This pattern holds across all three testbeds and signal types, though the area differential is most pronounced for RSS Only measurements. This trade-off between spatial efficiency and coverage guarantee reflects an inherent characteristic of uncertainty quantification frameworks and should be carefully considered when selecting confidence levels and region geometries for practical deployment.

## 6. Conclusions

This paper introduces a robust WiFi-based indoor positioning framework that combines RTT and RSS measurements with conformal prediction to deliver accurate location estimates alongside statistically valid uncertainty quantification. Motivated by the need for reliable indoor positioning in real-world applications, the proposed method leverages both RTT and RSS signal types to overcome the limitations of traditional point estimate systems and provides meaningful confidence measures without strong modeling assumptions.

Experimental results across three diverse real-world testbeds show that the hybrid RTT-RSS approach consistently outperforms single-signal methods, achieving a positioning accuracy of 0.6 m in challenging building floor environments. Conformal prediction yields efficient, circular prediction regions that maintain desired coverage rates (90–95%) while being significantly smaller than those produced by RSS-only systems. The framework proves robust across varied settings, though some limitations remain, such as larger prediction regions in high-variability scenarios and the need for improved handling of dynamic environments.

Future research will focus on adaptive conformalisation, handling temporal changes, and integrating additional sensor modalities to further enhance accuracy and efficiency. In conclusion, this work demonstrates that the proposed framework delivers both reliable accuracy and uncertainty quantification, making it well-suited for deployment in real-world challenging indoor scenarios where trustworthy location information is essential.

## Figures and Tables

**Table 1:** The details of the utilised datasets.

Dataset Features	Building Floor	Office Room	Apartment
Area	92 × 15 m^2^	5.5 × 4.5 m^2^	7.7 × 9.4 m^2^
Grid size	0.6 × 0.6 m^2^	0.455 × 0.455 m^2^	0.48 × 0.48 m^2^
Reference points	642	37	110
Samples per RP	120	120	120
Data samples	77,040	4440	13,200
Training samples	57,960	3240	9720
Testing samples	19,080	1200	3480
Signal measure	WiFi RTT, WiFi RSS	WiFi RTT, WiFi RSS	WiFi RTT, WiFi RSS
Other information	LoS condition of every AP	LoS condition of every AP	LoS condition of every AP
Collection time	3 days	1 day	1 day
Notes	A complex real-world scenario with both LoS and NLoS conditions	A LoS scenario	Contains an AP with NLoS paths for most of the RPs

**Table 2:** Device Specifications of Google WiFi Point.

Specification	Details
Bluetooth	Bluetooth Low Energy
Performance	Each point can handle up to 100 connected devices. Supports multiple simultaneous 4K video streams.

**Table 3:** Device Specifications of LG G8X ThinQ.

Specification	Details
OS	Android 9.0 (Pie)
SoC	Qualcomm Snapdragon 855 (SM8150) (Qualcomm Technologies, Inc., San Diego, CA, USA)
CPU	Octa-core, Qualcomm SDM855 Snapdragon 855 (7 nm) (Qualcomm Technologies, Inc., San Diego, CA, USA)
GPU	Qualcomm Adreno 640, 585 MHz (Qualcomm Technologies, Inc., San Diego, CA, USA)
RAM	6 GB, 2133 MHz
Storage	128 GB
Camera	4032 × 3024 pixels, 3840 × 2160 pixels, 30 fps
WiFi	WiFi 802.11 a/b/g/n/ac, Dual band, WiFi Hotspot, WiFi Direct, DLNA
USB	2.0, USB Type-C
Bluetooth	5.0
Positioning	GPS, A-GPS, GLONASS, Galileo
Security	WPA3 encryption, Automatic security updates, Trusted Platform Module (TPM)
WiFi Standard	AC1200 MU-MIMO WiFi (Qualcomm Technologies, Inc., San Diego, CA, USA). Simultaneous dual-band (2.4 GHz/5 GHz) supporting IEEE 802.11a/b/g/n/ac.
Processor	Quad-core ARM
Memory	512 MB RAM, 4 GB eMMC flash storage
Ports	Dual Gigabit Ethernet ports
Power	15 W power adaptor

**Table 4:** A Snapshot of the proposed WiFi dataset. The value −200 dBm in (**a**) and 100,000 mm in (**b**) indicate that the WiFi signals from the AP are not heard from the current reference point.

(**a**) WiFi RSS data samples						
**X**	**Y**	**AP1 RSS (dBm)**	**AP2 RSS (dBm)**	**…**	**AP13 RSS (dBm)**	**LoS APs**
1	15	−200	−200	…	−73	12
1	16	−200	−200	…	−70	12
2	0	−200	−200	…	−71	None
2	1	−200	−200	…	−63	12
…	…	…	…	…	…	…
125	15	−74	−47	…	−200	2 3
(**b**) WiFi RTT data samples						
**X**	**Y**	**AP1 RTT (mm)**	**AP2 RTT (mm)**	**…**	**AP13 RTT (mm)**	**LoS APs**
1	15	100,000	100,000	…	5958	12
1	16	100,000	100,000	…	4893	12
2	0	100,000	100,000	…	8716	None
2	1	100,000	100,000	…	10,062	12
…	…	…	…	…	…	…
125	15	10,585	598	…	100,000	23

**Table 5:** Positioning performance (in metres) of WiFi-based indoor positioning systems using different WiFi signal measurements. It is observed that WiFi RTT-RSS fingerprinting delivers the best positioning estimation.

Testbed	RTT + RSS	RTT	RSS
Building floor	0.60	0.70	1.38
Apartment	0.59	0.59	1.27
Office room	0.38	0.40	0.63

**Table 6:** The prediction intervals and coverage rates for reliable WiFi-based indoor positioning systems across different testbeds. ’Confidence’ is the user specified confidence level for conformal prediction. ’$q_{x}$’ and ’$q_{y}$’ are the prediction *q*-values for the $\hat{x}$ coordinate and $\hat{y}$ coordinate, respectively. It is observed that predicting $\hat{x}$ and $\hat{y}$ coordinates separately successfully meets the expected coverage rate.

(**a**) Building Floor					
**WiFi signal measures**	**Confidence**	**$q_{x}$ (m)**	**$q_{x}$ Coverage**	**$q_{y}$ (m)**	**$q_{y}$ Coverage**
RTT and RSS hybrid	90%	**0.79**	89.99%	**0.98**	90.22%
RTT and RSS hybrid	95%	1.03	95.23%	1.16	95.19%
RTT Only	90%	0.95	90.14%	1.15	89.92%
RTT Only	95%	1.13	94.85%	1.57	94.87%
RSS Only	90%	2.83	90.09%	1.51	90.09%
RSS Only	95%	3.65	95.22%	2.21	95.34%
(**b**) Apartment					
**WiFi signal measures**	**Confidence**	**$q_{x}$ (m)**	**$q_{x}$ Coverage**	**$q_{y}$ (m)**	**$q_{y}$ Coverage**
RTT and RSS hybrid	90%	**0.98**	89.43%	**0.72**	89.43%
RTT and RSS hybrid	95%	1.47	94.94%	0.86	95.06%
RTT Only	90%	1.06	90.75%	0.73	89.43%
RTT Only	95%	1.38	95.92%	0.99	95.80%
RSS Only	90%	2.79	91.38%	1.75	90.11%
RSS Only	95%	2.99	96.15%	1.97	94.83%
(**c**) Office Room					
**WiFi signal measures**	**Confidence**	**$q_{x}$ (m)**	**$q_{x}$ Coverage**	**$q_{y}$ (m)**	**$q_{y}$ Coverage**
RTT and RSS hybrid	90%	**0.50**	89.44%	**0.68**	93.33%
RTT and RSS hybrid	95%	0.82	94.26%	0.82	95.93%
RTT Only	90%	0.66	90.19%	0.77	90.37%
RTT Only	95%	0.67	97.59%	0.81	96.11%
RSS Only	90%	1.01	90.37%	1.77	90.00%
RSS Only	95%	1.15	95.00%	1.88	95.00%

**Table 7:** The prediction regions and coverage rates for reliable WiFi-based indoor positioning systems across different testbeds. ’Confidence’ is the user specified confidence level for conformal prediction. ‘$q_{p}$’ is the prediction *q*-value for the overall positioning $\left(\hat{x},\hat{y}\right)$. 2D coverage is the coverage rate calculated from the rectangular prediction region of $C_{rect}=\{\left(x^{\prime },y^{\prime }\right)|x^{\prime }\in \left[\hat{x}-q_{x},\;\hat{x}+q_{x}\right],$ $y^{\prime }\in \left[\hat{y}-q_{y},\;\hat{y}+q_{y}\right]\}$. ‘$q_{p}$ Coverage’ is the coverage rate calculated from the circular prediction region $C_{circ}=\left\{\left(x^{\prime },y^{\prime }\right)|\sqrt{{\left(x^{\prime }-\hat{x}\right)}^{2}+{\left(y^{\prime }-\hat{y}\right)}^{2}}\leq q_{p}\right\}$. It is observed that while circular regions provide coverage rates closer to the specified confidence levels, rectangular regions often yield smaller areas but with significantly lower 2D coverage rates, suggesting a trade-off between efficiency and coverage rage.

(**a**) Building Floor				
**WiFi signal measures**	**Confidence**	**2D Coverage**	**$q_{p}$ (m)**	**$q_{p}$ Coverage**
RTT and RSS hybrid	90%	81.75%	**1.20**	90.01%
RTT and RSS hybrid	95%	90.68%	1.45	95.39%
RTT Only	90%	82.75%	1.37	89.79%
RTT Only	95%	90.89%	1.84	94.96%
RSS Only	90%	81.48%	3.16	90.16%
RSS Only	95%	90.90%	3.97	95.29%
(**b**) Apartment				
**WiFi signal measures**	**Confidence**	**2D Coverage**	**$q_{p}$ (m)**	**$q_{p}$ Coverage**
RTT and RSS hybrid	90%	80.52%	**1.11**	90.52%
RTT and RSS hybrid	95%	90.17%	1.52	95.52%
RTT Only	90%	82.59%	1.29	89.94%
RTT Only	95%	91.90%	1.79	96.26%
RSS Only	90%	85.63%	3.27	90.52%
RSS Only	95%	92.36%	3.68	94.77%
(**c**) Office Room				
**WiFi signal measures**	**Confidence**	**2D Coverage**	**$q_{p}$ (m)**	**$q_{p}$ Coverage**
RTT and RSS hybrid	90%	84.07%	0.90	90.56%
RTT and RSS hybrid	95%	90.56%	0.96	96.30%
RTT Only	90%	80.56%	**0.86**	89.26%
RTT Only	95%	93.70%	0.89	95.93%
RSS Only	90%	80.37%	1.88	93.15%
RSS Only	95%	90.00%	1.97	99.44%

**Table 8:** Comparison of prediction region areas and coverage rates for WiFi-based indoor positioning systems. The rectangular prediction region is defined as $C_{\mathrm{rect}}=\{\left(x^{\prime },y^{\prime }\right)|x^{\prime }\in \left[\hat{x}-q_{x},\hat{x}+q_{x}\right],$ $y^{\prime }\in \left[\hat{y}-q_{y},\hat{y}+q_{y}\right]\}$ with area $4\times q_{x}\times q_{y}$. The circular prediction region is defined as $C_{\mathrm{circ}}=\left\{\left(x^{\prime },y^{\prime }\right)|\sqrt{{\left(x^{\prime }-\hat{x}\right)}^{2}+{\left(y^{\prime }-\hat{y}\right)}^{2}}\leq q_{p}\right\}$ with area $\pi \times q_{p}^{2}$. It is observed that under identical signal measures and confidence levels, smaller prediction regions with maintained coverage indicate higher efficiency and reliability. The analysis reveals that while circular regions provide coverage rates closer to the specified confidence levels, rectangular regions often yield smaller areas but with significantly lower 2D coverage rates, suggesting a trade-off between efficiency and coverage rage.

(**a**) Building Floor					
**WiFi signal measures**	**Confidence**	**Rectangular Area (m^2^)**	**2D Coverage**	**Circular Area (m^2^)**	**$q_{p}$ Coverage**
RTT and RSS hybrid	90%	3.10	81.75%	4.52	90.01%
RTT and RSS hybrid	95%	4.78	90.68%	6.60	95.39%
(**b**) Apartment					
**WiFi signal measures**	**Confidence**	**Rectangular Area (m^2^)**	**2D Coverage**	**Circular Area (m^2^)**	**$q_{p}$ Coverage**
RTT and RSS hybrid	90%	2.82	80.52%	3.87	90.52%
RTT and RSS hybrid	95%	5.06	90.17%	7.26	95.52%
(**c**) Office Room					
**WiFi signal measures**	**Confidence**	**Rectangular Area (m^2^)**	**2D Coverage**	**Circular Area (m^2^)**	**$q_{p}$ Coverage**
RTT and RSS hybrid	90%	1.36	84.07%	2.54	90.56%
RTT and RSS hybrid	95%	2.69	90.56%	2.90	96.30%

## Data Availability

The data presented in this study are openly available in Zenodo at https://doi.org/10.5281/zenodo.11558192.
